# The psychological impact of COVID-19 in a socio-politically unstable environment: protective effects of sleep and gratitude in Lebanese adults

**DOI:** 10.1186/s40359-023-01042-4

**Published:** 2023-01-19

**Authors:** Myriam El Khoury-Malhame, Rana Rizk, Esperance Joukayem, Alyssa Rechdan, Toni Sawma

**Affiliations:** 1 Department of Social and Education Sciences, School of Arts and Sciences, Lebanese American University, Beirut, Lebanon; 2 Department of Natural Sciences, School of Arts and Sciences, Lebanese American University, Beirut, Lebanon; 3 INSPECT-LB, (Institut National de Santé Publique, d’Epidémiologie Clinique Et de Toxicologie-Liban), Beirut, Lebanon

**Keywords:** COVID-19, PTSD, Anxiety, Depression, Perceived stress, Gratitude, Sleep

## Abstract

**Background:**

The COVID-19 global pandemic initiated an unprecedented medico-psychological turmoil. Our study investigates the psychological impact of the viral spread and austere lockdown, and focuses mostly on potential protective factors in a politically and economically unstable society.

**Methods:**

A cross-sectional design was used to evaluate rates of mental distress in a sample of 348 Lebanese adults. Participants filled questionnaires of perceived stress (PSS), depression (PHQ-9), anxiety (GAD-7), PTSD (IES-22), as well as sleep (PSQi) and gratitude (GQ-6) immediately after 3 months of strict quarantine. Demographics included gender, age, employment and infection statuses. Correlations and regression models were used.

**Results:**

Results indicated a very high prevalence of mental distress, in addition to major alterations in sleep quality and quantity. Younger age and unemployment correlated with more severe symptoms. Sleep was found to be a protective factor against all studied psychological distress, and gratitude further mitigated effects of depression.

**Conclusions:**

Mental health significantly degrades post-COVID lockdown, even in the absence of direct viral threat. Yet simple behavioral and cognitive changes like sleep and attitude of gratitude could provide protective factors against these psychological distresses. Such changes should be further explored and advocated as cost-efficient self-care practices to buffer this devastating public health burden, especially in unstable socio-political environments.

## Background

The Corona Virus Disease 2019 (COVID-19), a lethal strand of pathogenic human coronavirus caused by the caused by the Severe Acute Respiratory Syndrome Coronavirus 2 (SARS-CoV-2 virus), spread from Wuhan China in December 2019 and was soon after declared a pandemic on March 2020 by the World Health Organization (WHO). Public health instances rapidly advocated restrictive lockdowns, suspension of global traveling, and strict sanitization procedures in addition to social distancing [1]. The dangerous viral spread was reported to heavily impact overall physical and mental well-being, noting drastic behavioral and psychological consequences, accompanied by health concerns [2]. Clinically, symptomatic COVID-19 patients reported symptoms ranging from mild to high fever and coughing [3], all the way to pulmonary edema, severe pneumonia, respiratory constriction and subsequent organ failures ultimately leading to death [4]. The medical contagion curve paralleled psychological distress with most people experiencing fear of contamination, intolerance of uncertainty, anger, and insomnia [2]. During the initial viral spread, more than 50% of respondents in China for instance indicated a moderate to severe psychological impact with one third scoring reporting moderate to severe anxiety and depression [5].

The past pandemics have shown that prolonged lockdown further aggravates the toll on psychological health [6]. After the SARS outbreaks in 2003, a strong positive correlation was observed between fear of contamination and post-traumatic stress disorder (PTSD) even among recovered patients [6]. Fighting a global unprecedented virus provokes major life-threatening shift in quality of life resembling physical and mental complications experienced in the aftermath of traumatic events [7–9]. Recent reviews and meta-analyses showed that chronic fear of COVID-19 infection and generalized worries alongside strict lockdown measures exacerbated psychological distress and led to increased rates of depression, anxiety, and PTSD [3, 10]. Longer confinement periods correlated with poorer mental health and higher incidences of PTSD [11] whereas lengthy quarantine increased loneliness, irritability and insomnia [6], further aggravating the human and financial costs of such an outbreak [12].

In such global pandemics, with tremendous infrastructural demands, limited medico-psychological resources and underequipped health facilities [13], identifying vulnerable individuals becomes crucial. Although men are twice as likely as women to be infected, hospitalized, and die of COVID-19 [14], women are at higher risk of suffering from mental distress [15], especially during the prolonged quarantine [16]. People below the age of 25–35 or those who have a low educational level [17] experience the pandemic as being significantly more stressful. Studies after SARS infections report that medical employees, directly or indirectly caring got infected patients were twice to thrice more likely to develop PTSD than other employees [18]. A more recent study of around 1250 health professionals in China caring for COVID-19 patients, found they had elevated levels of anxiety, PTSD, depression, and insomnia [19].

Preliminary reports also argue that the profound changes in individual health behaviors including sleep, physical activity, diet, and substance use would lead to damaging downstream mental health consequences during strict quarantine [20]. Understanding population level changes in health behaviors remains understudied and should be better investigated to apprehend associated costs and potentially optimize management measures. In fact, some cost-efficient lifestyle health behaviors like adequate sleep patterns and physical activity are known to positively correlate with healthier mental functioning [15]. For instance, more efficient sleeping patterns were associated with an overall improved mental and physical well-being [21] and complaints about anxiety and somatic symptoms were diminished in students with healthy nighttime sleep [0]. On the contrary, a global decline in sleep hours was observed in line with the rise of lifestyle-driven chronic diseases [22] and mental concerns [23]. Counter-intuitively, although people spend more time at home, the COVID-19 lockdown was associated with delayed onset of sleep and reduced quality of sleep [24]. Those sleep alterations in turn worsen onset and prognosis of mental distress [25]. A UK study on 2250 participants revealed 38% of respondents had decreased sleep quality and quantity since the beginning of the pandemic, and 49% of them felt more anxious and depressed [26]. Another factor known to mitigate negative mental health impact is gratitude. Gratitude can be viewed as an optimistic affective reaction to positive outcomes in life [27]. Coping with difficult times can be facilitated through faith and gratitude [28]. The daily practice of gratitude is generally known to correlate with improved well-being and decreased incidences of suicidal thoughts, physical complaints [28], PTSD, and depression [27]. During the pandemic, grateful people reported reduced focus on the disagreeable life situations due to COVID-19, had lower stress levels, and better perceived health indices [28].

On February 21st, Lebanon documented its first case of coronavirus and quickly declared a state of three months of complete lockdown [29]. Lebanon is a small middle-eastern country faced with semi-continuous conflict for the past few decades, with a notable socio-economic crisis lingering since October 2019 [30]. The collapse of the local currency and shortage of imported goods is worsened by the fact that this country of 6 million inhabitants host more than 1.5 million Syrian refugees. Similar to other countries on strict sanitary regulations, the population showed elevated levels of anxiety and depression in correlation with social isolation [20]. The confinement additionally led to increased domestic violence and job loss in a country challenged with financial frailty and sociopolitical unrest [31]. Job insecurities seemingly worsened mental health as it contributed to increased depressive and anxious symptoms [32].

Considering COVID-19 spread threatens both physical and psychological health, it is particularly thought-provoking to better understand the psychological implications of its subsequent lockdown in a population faced with collective challenges. On the other hand, Lebanon is known for the elevated prevalence of spirituality and religiosity in its adult population [33], conducive to potentially promoting gratitude, in spite of hardships, as a potential protective factor.

As such, our study first and foremost aims at assessing psychological distress in an unstable society hypothesizing it will vary with age, as younger adults might be more vulnerable, with gender, as females would show higher symptomatic intensity, and with job status, as job uncertainty seems to be an additional risk factor for mental distress. We also investigate whether sleep quality and quantity in addition to gratitude would protect Lebanese adults against perceived stress, anxiety, depression, and PTSD amidst the pandemic. These would provide valuable recommendations to inform public health policies in Lebanon and beyond to better mitigate the overall increased levels of mental distress.

## Methods

### Participants

Initially a total of 360 adult participants agreed to participate in the study. However, 12 of them had their data discarded due to either stopping mid-way through and exiting the survey (6 participants) and 6 others were not within the appropriate age range. The final sample included 348 adults whose socio-demographic data are presented in Table 1.

**Table 1:** Socio-demographic data of the sample (N = 348)

Variable	N [%]
Gender	
Male	98 [28.16%]
Female	250 [71.84%]
Education level	
Secondary or less	72 [20.69%]
University	276 [79.31%]
Healthcare provider	
No	289 [83.05%]
Yes	59 [16.95%]
Professional status	
Unemployed	207 [59.48%]
Employed	141 [40.52%]
Job loss	
No	166 [81.37%]
Yes	38 [18.63%]
Not applicable	144 [41.4%]
COVID-19 infection during the lockdown	
No	334 [95.98%]
Yes	14 [4.02%]
Knows anyone who got infected by COVID-19	
No	219 [62.93%]
Yes	129 [37.07%]
Changed residency due to COVID-19	
No	301 [86.49%]
Yes	46 [13.22%]

### Procedure

The study was done in according to the Declaration of Helsinki and approved by the Institutional Review Board (IRB) board of the Lebanese American University (LAU) [LAU.SAS.MM11.18/May/2020]. All participants approved the informed consent before filling the 10–15 min questionnaire and demographics on a circulated Google link, disseminated via email and other social media platforms such as LinkedIn, Facebook, and WhatsApp between the months of May and June 2020 at the end of 3 months of extremely strict lockdown imposed in Lebanon. Sampling was based on convenience and snowballing techniques to include participants from various regions residing in Lebanon.

### Measurements and scales

Six psychological self-filled scales were used to assess the psychological variables and standard cut-off levels for severity were used, respectively.

- 1. Perceived Stress Scale [PSS] is a ten-item self-report measure to measure the degree to which situations in one’s life are assessed as stressful. Originally developed by Cohen et al. (1983) it is widely used to assess stress levels by asking about feelings and thoughts relating to personal perceptions of events in the past month. Items include statements such as “In the last month, how often have you been upset because of something that happened unexpectedly?” and “In the last month, how often have you found that you could not cope with all the things that you had to do?”. These statements are rated from 0 to 4 according to the following alternatives: never, almost never, sometimes, fairly often, and very often. Scores ranging from 0 to 13 are considered low stress, from 14 to 26 moderate stress, and from 27 to 40 high stress. PSS has a high internal consistency reliability and factorial validity [27]. Its documented Cronbach alpha typically ranges between 0.74 and 0.91, with a value of 0.81 for the current study.
- 2. Generalized anxiety disorder [GAD-7] is a seven-item self-report used to detect and measure severity of generalized anxiety. Items include statements such as “Feeling anxious, nervous or on edge” rated for intensity of occurrence over the last two weeks from 0 to 3 respectively for the following options: not at all, several days, over half the days, or nearly every day. A cutoff value of ≥ 5 was used as indication of mild to moderate anxiety and a value of > 15 indicated severe anxiety in our study. The scale has a Cronbach’s alpha of 0.89 for internal consistency [34], and a value of 0.91 in the current study.
- 3. Patient Health Questionnaire [PHQ-9] is a nine-item self-report measure to monitor the presence and severity of depression symptoms. Items include statements such as “Feeling down, depressed or helpless” and “Little pleasure or interest in doing things” rated for intensity of occurrence over the last two weeks from 0 to 3 respectively for the following options: not at all, several days, more than half the days, or nearly every day. A cutoff value of ≥ 5 was used as indication of mild to moderate depression and a value of > 20 indicated severe depression in our study. The scale has shown robust reliability and validity [Cronbach alpha = 0.85–0.91] [35], with a value of 0.87 in the current study.
- 4. Impact of Event Scale [IES-22] is one of the most widely-used self-report measures within the trauma literature, with Cronbach alpha ranging from 0.84 to 0.91[36] [α = 0.94]. It is a 22-item scale used to evaluate the degree of distress one experiences in response to a given trauma. Items include statements such as “any reminder brought back feelings about it”, “I had trouble concentrating” and “I tried not to talk about it”. Items are rated for distressing levels over the last seven days on a scale from 0 [not at all] to 4 [extremely]. A cutoff value of > 24 was used as indication of clinical worry and a value of > 33 indicated likely PTSD diagnosis in our study [36].
- 5. Pittsburgh Sleep Quality Index [PSQI] consists of 18 items on a four-point Likert scale, and is designed to measure sleep disturbances and sleep habits over a one-month period. It includes questions about time of bed, the number of hours of sleep per night, wake up time, and the time it takes to fall asleep. It also includes statements such as “during the past month how often have you had trouble sleeping because you have bad dreams” and “how would you rate your overall quality of sleep”. Each statement is scored between 0 [not during the past month] and 3 [three or more times a week]. Statements are broken down into seven components and converted to a point score. Higher scores indicate poorer sleep hygiene and scores > 5 point to poor sleep quality. Validity and reliability have been previously reported [37]. Participants were asked to report on their daily estimated sleep quantity and time to bed in hours. This scale has a documented Cronbach alpha of 0.83, similarly to the value reported in our sample [38].
- 6. Gratitude Questionnaire [GQ-6] is a six-item self-report measure designed to quantify individual variances in the proneness to experience gratitude in daily life. Items are rated on a seven-point Likert-type scale, where 1 = strongly disagree and 7 = strongly agree, and include statements like “I have so much in life to be thankful for” and “When I look at the world, I don't see much to be grateful for”. Total score ranges between 6 and 42, with higher scores indicating higher gratitude. The overall Cronbach alpha is 0.84 in validating studies [‎42] and 0.79 in the current study.

### Statistical analysis

All statistical analyses were conducted using the Statistical Package for Social Sciences (SPSS), version 25. Scores for stress [PSS], anxiety [GAD-7], depression [PHQ-9], PTSD [IES-22], sleep [PSQI], and gratitude [GQ] were visually inspected for outliers. PSS, GAD-7, PHQ-9, and IES-22 scores were considered as normally distributed as verified by the visual inspection of the histogram and values of skewness and kurtosis below |1.96|, although the Kolmogorov–Smirnov test of normality was significant (*p* < 0.05). A bivariate analysis was conducted using the Student’s independent t-test to compare continuous variables in two groups and the Analysis of Variance (ANOVA) test to compare three or more means. Pearson correlation was used for linear correlations between continuous variables. Since criteria for multivariate normality were met, four multivariate regression analyses using the “Enter” method were conducted to test if the independent variables of sleep and gratitude could predict mental health outcomes, i.e., anxiety, depression, PTSD, and stress while adjusting for age, gender, employment status, profession, and COVID-19 infection. The normality line of the regression plot and the scatter plot of the residuals verified the normality of the scales used. A *p* < 0.05 was considered statistically significant for all tests.

## Results

### Demographics

The mean age of the participants was 26 years (SD = 7.85), the majority were females (71.84%), have a university education level (79.31%), and unemployed (59.48%). At the time of the assessment, only 4% had been infected by COVID-19 during the lockdown; yet 37% knew someone who got infected by COVID-19. Among those, around 17% were healthcare providers with 13.22% having changed their residency because of the pandemic.

### Mental distress frequency

Mean and standard deviations for stress [PSS], anxiety [GAD-7], depression [PHQ-9], PTSD [IES-22], insomnia [PSQI], and gratitude [GQ-6] are presented in Table 2. Around 85% of participants scored above the cut-off for mental health distress, and the frequency rates for severe mental distress were 34.7% for stress, 27.9% for anxiety, 10.6% for depression, 44.5% for PTSD, and 50% for insomnia.

**Table 2:** Means and standard deviations of mental health variables

	M	SD	Median	Skewness	Kurtosis	Kolmogorov–Smirnov	
						d-statistic	*P* value
PSS	23.22	6.54	23.00	− 0.19	− 0.35	0.067	0.001
GAD-7	10.57	5.48	10.00	0.13	− 0.87	0.091	< 0.001
PHQ-9	11.17	6.33	10.00	0.51	− 0.34	0.092	< 0.001
IES-22	31.20	18.67	28.00	0.40	− 0.47	0.073	< 0.001
PSQi	6.15	3.47	5.50	0.77	0.53	0.131	< 0.001
GQ-6	31.88	6.74	33.00	− 0.61	− 0.08	0.097	< 0.001

Perceived Stress Scale [PSS], Generalized Anxiety Disorder [GAD-7], Patient Health Questionnaire [PHQ-9] and Impact of Events Scale [IES-22] as well as Pittsburg Sleep Quality Index [PSQI] and Gratitude Questionnaire [GQ-6]

### Bivariate analysis of mental distress

The results of the bivariate analysis taking the mental health scales as the dependent variables are shown in Tables 3 and 4.

**Table 3:** Bivariate analysis of categorical variables associated with depression and anxiety

	PHQ-9					GAD-7				
	M	SD	t	*p*	Cohen's d	M	SD	t	*p*	Cohen's d
Gender										
Female	11.26	6.56	− 0.43	0.663	0.05	10.63	5.36	− 0.36	0.713	0.04
Male	10.94	5.74				10.39	5.80			
Age group										
18–25	12.40	6.56	5.12	< 0.001	− 0.54	11.14	5.56	2.64	0.009	− 0.29
> 25	9.07	5.37				9.54	5.24			
Professional status										
Employed	10.10	5.86	2.61	0.009	− 0.28	10.23	5.37	0.946	0.345	− 0.10
Unemployed	11.89	6.55				10.80	5.55			

	M	SD	F (2, 347)	p	η^2^	M	SD	F (2, 346)	p	η^2^
Job loss										
Yes	14.15	6.14	10.44	< 0.001	0.72	11.73	5.18	3.73	0.025	0.28
No	9.72	5.95				9.75	5.37			
Not applicable	12.04	6.41				11.20	5.57			

Values for the M [mean] and SD [standard deviation], for the anxiety Generalized Anxiety Disorder [GAD-7], and depression Patient Health Questionnaire [PHQ-9] with their respective T-statistic, F-statistic, p-value for the various analysis

**Table 4:** Bivariate analysis of categorical variables associated with stress and PTSD

	PSS					IES-22				
	M	SD	t	p	Cohen's d	M	SD	t	p	Cohen's d
Gender										
Female	23.61	6.23	− 1.64	0.102	0.20	31.08	18.66	0.19	0.847	− 0.02
Male	22.24	7.21				31.51	18.76			
Age group										
18–25	24.04	6.92	3.30	0.001	− 0.35	33.40	19.18	2.99	0.003	− 0.33
> 25	21.77	5.67				27.29	17.33			
Professional status										
Employed	22.20	5.74	2.49	0.013	− 0.26	27.91	16.73	2.81	0.005	− 0.29
Unemployed	23.91	6.96				33.43	19.60			

	M	SD	F (2, 347)	p	η^2^	M	SD	F (2, 347)	p	η^2^
Job loss										
Yes	24.68	6.53	4.92	0.008	0.39	34.31	20.05	4.86	0.008	0.33
No	22.09	5.96				27.96	17.55			
Not applicable	24.13	6.99				34.10	19.06			

Values for the M [mean] and SD [standard deviation] for the stress Perceived Stress Scale [PSS], and PTSD Impact of Events Scale [IES-22] with their respective T-statistic, F-statistic, p-value for the various analysis

Results showed that age, professional status, and job loss were significantly associated with depression. Higher depression scores were found among younger unemployed participants and those who lost their jobs as compared with the others (Table 3).

Higher anxiety levels were documented among younger adults as compared with those over 25 years and in participants reporting a job loss v/s. the other groups (Table 3).

As for stress (PSS scale), age, professional status, and job loss significantly contributed. Once again, higher stress levels were identified among younger participants compared with those over 25 years, and among those unemployed or who had lost their jobs as compared with employed ones (Table 4).

Finally, results showed that PTSD symptomatology was higher in young adults and unemployed participants or those who reported job loss as compared with others (Table 4).

## Bivariate analysis of sleep and gratitude

The results showed that gender and job loss were significantly associated with insomnia. A poorer sleep quality was found among male participants as compared with females and in those who lost their jobs v/s. the other groups (Table 5).

**Table 5:** Bivariate analysis of categorical variables associated with sleep and gratitude

	PSQI					GQ-6				
	M	SD	t	p	Cohen's d	M	SD	t	p	Cohen's d
Gender										
Female	5.88	3.43	2.36	0.018	− 0.28	32.47	6.56	− 2.60	0.010	0.31
Male	6.85	3.47				30.39	7.00			
Age group										
18–25	6.29	3.55	0.94	0.347	− 0.10	30.98	7.19	− 3.28	0.001	0.34
> 25	5.93	3.36				33.30	5.80			
Professional status										
Employed	5.79	3.23	1.61	0.107	− 0.17	32.58	6.31	− 1.61	0.107	0.17
Unemployed	6.40	3.60				31.41	7.00			

	M	SD	F (2, 347)	p	η^2^	M	SD	F (2, 347)	p	η^2^
Job loss										
Yes	6.86	3.68	3.41	0.034	0.33	32.86	6.23	1.75	0.174	0.28
No	5.66	3.24				32.34	6.34			
Not applicable	6.54	3.60				31.10	7.27			

Values for the M [mean] and SD [standard deviation] for the various scales Pittsburg Sleep Quality Index [PSQI] and Gratitude Questionnaire [GQ-6] with their respective T-statistic, F-statistic, p-value for the various analysis

As for gratitude, the results showed that female gender and younger age were significantly associated with its higher levels (Table 5).

### Sleep and gratitude in relation to mental distress

Table 6 illustrates sleep and gratitude as a function of mental health variables. The results show that subjects with higher PSQI scores, i.e., worst sleep quality, scored significantly higher than the others for all mental distress variables, including PHQ-9, GAD-7, PSS, and IES-22.

**Table 6:** Breakdown on sleep and gratitude means according to mental health variables

	PHQ-9			GAD-7			PSS			IES-22		
	M (SD)	t	Cohen’s D	M (SD)	t	Cohen’s D	M (SD)	t	Cohen’s D	M (SD)	t	Cohen’s D
PSQI												
< 5 (n = 174)	8.45 (5.39)	− 8.85***	0.95	8.44 (5.04)	− 7.82***	0.84	21.65 (6.08)	− 4.62***	0.49	24.13 (16.56)	− 7.61***	0.81
> 5 (n = 174)	13.89 (6.05)			12.68 (5.07)			24.80 (6.62)			38.26 (18.01)		
GQ-6												
< 33 (n = 187)	12.79 (6.21)	5.33***	− 0.57	11.94 (5.08)	5.22***	− 0.56	24.87 (6.13)	5.28***	− 0.57	33.98 (18.95)	3.03***	− 0.32
> 33 (n = 161)	9.29 (5.96)			8.97 (5.51)			21.28 (6.49)			27.96 (17.85)		

Values for the M [mean] and SD [standard deviation] for the various scales Pittsburg Sleep Quality Index [PSQI] and Gratitude Questionnaire [GQ-6] with their respective T-statistic for the various mental health variables including Generalized Anxiety Disorder [GAD-7], and Patient Health Questionnaire [PHQ-9], Perceived Stress Scale [PSS], and Impact of Events Scale [IES-22]

P values indicated as ***p < 0.001

As for GQ-6, subjects with higher scores, i.e., better gratitude attitude, scored lower than the others for all mental distress variables, including PHQ-9, GAD-7, PSS, and IES-22.

### Correlations between mental distress and sleep and gratitude

The correlation matrix is presented in Table 7. PSQI was significantly positively correlated with all mental health factors indicating a lower quality of sleep is associated with higher scores of stress, depression, anxiety and trauma; whereas gratitude was significantly negatively correlated with all mental health indicators.

**Table 7:** Correlational values between mental distress, sleep and gratitude

	PSS	GAD-7	PHQ-9	IES-22	PSQi	GQ-6
PSS	–	0.73***	0.65***	0.56***	0.34***	− 0.35***
GAD-7		–	0.72***	0.65***	0.46***	− 0.32***
PHQ-9			–	0.65***	0.51***	− 0.37***
IES-22				–	0.49***	− 0.26***
PSQi					–	− 0.29***
GQ-6						–

Pearson Correlation r-values for the variables of Perceived Stress Scale [PSS], Generalized Anxiety Disorder [GAD-7], Patient Health Questionnaire [PHQ-9] and Impact of Events Scale [IES-22] as well as Pittsburg Sleep Quality Index [PSQI] and Gratitude Questionnaire [GQ-6]

p-values indicated as ***p < 0.001

Gratitude was further negatively correlated to altered sleep quality, as more grateful people seem to have lower alterations in sleep quality.

### Multiple regression model

The results of the four regression analyses indicate that sleep and gratitude are protective factors for anxiety, depression, stress, and PTSD.

First considering depression, results indicated 40.07% of its variance was accounted for by poorer sleep quality (β = 0.81, *p* < 0.001), being a female (β = 1.56, *p* = 0.010) and having lost one’s job (β = 2.72, *p* = 0.002). However, being over 25 years (β = − 2.55, *p* < 0.001), being a healthcare provider (β = − 1.60, *p* = 0.031) and reporting higher gratitude (β = − 0.23, *p* < 0.001) were associated with lower depression (Table 8).

**Table 8:** Multivariate analysis: linear regression with depression as the dependent variable

	B (SE)	95% CI	β	t	p
Job loss (yes vs no^a^)	2.72 (0.87)	1.01; 4.44	0.13	3.13	0.002
Professional (employed vs unemployed^a^)	0.78 (0.65)	− 0.50; 2.06	0.06	1.19	0.232
Gender (female vs male^a^)	1.56 (0.60)	0.38; 2.75	0.11	2.60	0.010
Age (> 25 vs 18–25^a^ years)	− 2.55 (0.64)	− 3.82; − 1.27	− 0.19	− 3.93	< 0.001
Health care provider (yes vs no^a^)	− 1.60 (0.74)	− 3.06; − 0.14	− 0.09	− 2.16	0.031
Education cat (university vs school^a^)	− 0.24 (0.73)	− 1.68; 1.19	− 0.01	− 0.33	0.740
Gratitude (GQ-6)	− 0.23 (0.04)	− 0.32; − 0.15	− 0.25	− 5.47	< 0.001
Insomnia (PSQI)	0.81 (0.08)	0.65; 0.97	0.44	9.99	< 0.001

Pittsburg Sleep Quality Index [PSQI] and Gratitude Questionnaire [GQ-6]

B = unstandardized beta; SE = Standard error; β = standardized beta; CI = Confidence interval; T-statistic and p-value

^a^Reference group

As for anxiety, predictors taken together point to poorer sleep quality (β = 0.65, *p* < 0.001) and being a female (β = 1.17, *p* = 0.042) as associated with higher anxiety; whereas being older (β = − 1.37, *p* = 0.027) and having higher gratitude (β = − 0.17, *p* < 0.001) significantly associated with lower anxiety (Table 9).

**Table 9:** Multivariate analysis: linear regression with anxiety as the dependent variable

	B (SE)	95% CI	β	t	p
Job loss (yes vs no^a^)	0.96 (0.83)	− 0.66; 2.60	0.05	1.16	0.245
Professional (employed vs unemployed^a^)	0.88 (0.62)	− 0.34; 2.11	0.08	1.42	0.156
Gender (female vs male^a^)	1.17 (0.57)	0.04; 2.29	0.09	2.04	0.042
Age (> 25 vs 18–25^a^ years)	− 1.37 (0.62)	− 2.59; − 0.16	− 0.12	− 2.22	0.027
Health care provider (yes vs no^a^)	− 0.48 (0.70)	− 1.87; 0.90	− 0.03	− 0.68	0.494
Education cat (university vs school^a^)	0.16 (0.69)	− 1.20; 1.54	0.01	0.24	0.808
Gratitude (GQ-6)	− 0.17 (0.04)	− 0.25; − 0.09	− 0.21	− 4.22	< 0.001
Insomnia (PSQI)	0.65 (0.07)	0.49; 0.80	0.41	8.41	< 0.001

Pittsburg Sleep Quality Index [PSQI] and Gratitude Questionnaire [GQ-6]

B = unstandardized beta; SE = Standard error; β = standardized beta; CI = Confidence interval; T-statistic and *p* value

^a^Reference group

The stress model was predicted by poorer sleep quality (β = 0.50, *p* < 0.001) and being a female (β = 2.37, *p* = 0.001) with higher gratitude (β = − 0.29, *p* < 0.001) significantly associated with lower stress (Table 10).

**Table 10:** Multivariate analysis: linear regression with stress as the dependent variable

	B (SE)	95% CI	β	t	*p*
Job loss (yes vs no^a^)	1.27 (1.01)	− 0.72; 3.26	0.06	1.25	0.210
Professional (employed vs unemployed^a^)	− 0.23 (0.75)	− 1.72; 1.26	− 0.01	− 0.30	0.760
Gender (female vs male^a^)	2.37 (0.70)	0.99; 3.75	0.16	3.38	0.001
Age (> 25 vs 18–25^a^ years)	− 1.24 (0.75)	− 2.73; 0.23	− 0.09	− 1.65	0.098
Health care provider (yes vs no^a^)	− 1.47 (0.86)	− 3.17; 0.22	− 0.08	− 1.70	0.089
Education cat (university vs school^a^)	0.77 (0.85)	− 0.91; 2.45	0.04	0.90	0.367
Gratitude (GQ-6)	− 0.29 (0.05)	− 0.39; − 0.19	− 0.30	− 5.92	< 0.001
Insomnia (PSQI)	0.50 (0.09)	0.32; 0.69	0.27	5.37	< 0.001

Pittsburg Sleep Quality Index [PSQI] and Gratitude Questionnaire [GQ-6]

B = unstandardized beta; β = standardized beta; CI = confidence interval; T-statistic and *p* value

^a^Reference group

Finally, results indicated that poorer sleep quality (β = 2.47, *p* < 0.001) predicted increased PTSD symptoms while being a healthcare provider (β = − 7.82, *p* = 0.001) and having higher gratitude (β = − 0.36, *p* = 0.007) predicted lower symptoms (Table 11).

**Table 11:** Multivariate analysis: linear regression with PTSD as the dependent variable

	B (SE)	95% CI	β	T	*p*
Job loss (yes vs no^a^)	1.15 (2.77)	− 4.30; 6.60	0.02	0.41	0.678
Professional (employed vs unemployed^a^)	0.32 (2.07)	− 3.75; 4.39	0.01	0.15	0.877
Gender (female vs male^a^)	2.72 (1.91)	− 1.04; 6.48	0.06	1.42	0.156
Age (> 25 vs 18–25^a^ years)	− 2.70 (2.06)	− 6.75; 1.34	− 0.07	− 1.31	0.190
Health care provider (yes vs no^a^)	− 7.82 (2.35)	− 12.46; − 3.18	− 0.15	− 3.31	0.001
Education cat (university vs school^a^)	− 3.86 (2.32)	− 8.44; 0.70	− 0.08	− 1.66	0.097
Gratitude (GQ-6)	− 0.36 (0.13)	− 0.63; − 0.09	− 0.13	− 2.69	0.007
Insomnia (PSQI)	2.47 (0.25)	1.96; 2.97	0.46	9.56	< 0.001

Pittsburg Sleep Quality Index [PSQI] and Gratitude Questionnaire [GQ-6]

B = unstandardized beta; β = standardized beta; CI = confidence interval; T-statistic and *p* value

^a^Reference group

## Discussion

Wearing masks and socially distancing measures decreased risk of contamination, yet did not halt the propagation of worries and emotional distress related to COVID-19. This study assessed the psychological impact of COVID-19 prolonged quarantine in a convenient Lebanese sample during times of political turmoil and civil unrest. It further investigated the predictive value of gratitude and sleep in mitigating these deleterious outcomes.

Our study first and foremost indicates extremely elevated levels of mental distress, including perceived stress, depression, anxiety, and PTSD, independent of COVID-19-infection status. An alarming 85% of individuals score above detectable cut-off for stress, anxiety, and depression with over one third of them reporting severe symptoms and 60% having all three comorbidities altogether. Rates detected are similar to other studies on the same population [39, 40] and remain somewhat higher than those documented with the COVID-19 pandemic in general [41], in health care providers [42], and even in COVID-19-recovered patients [43], whereby figures approximate that around 30–50% of people struggled with stress, anxiety, and depression [44]. This could be accounted for by the lurking sociopolitical and economic insecurities in Beirut as preliminary studies point to the interplay between fear of COVID-19 and financial wellness in increasing mental hardship in Lebanon [45] further inflating the pre-pandemic reports of one third of Lebanese adults struggling with psychological distress [46]. Results are to be taken with caution as to their longitudinal implications since systematic reviews and meta-analyses show rates of anxiety and depression are quite similar between various professions and countries [47], and are systematically higher than pre-pandemic global prevalence rates [48]. This considerable degree of initial overall psychological distress and behavioral alterations could as such be a reflection of the temporary cost of adjustment to abnormal situations, rather than an indicator of psychiatric disorders [49].

The pandemic and its subsequent strict three-month lockdown were perceived as a major trauma in this study. Indeed, 60% of participants displaying clinically worrisome COVID-19-related trauma symptoms, in line with meta-analysis results evidencing PTSD as the main mental distress in times of pandemic [10], with worrying indications that its levels are higher than those reported in collective traumas such as hurricanes and terrorist attacks [50]. This highlights the significant short and long-term impact of COVID-19 on mental health in the global community and more specifically points to the necessity to focus on countries with pre-existing national struggles and piling up traumas [51].

Gender data was controversial in COVID-19 outcomes in the general population [16]. We did not find differences in rates of anxiety, depression, nor PTSD between males and females suggesting increased overall severity of mental distress among both subgroups [47]. However, younger age, job loss, and unemployment status were found to score higher than other demographic categories on all aforementioned measures of mental disorders, similarly to previously published data [52]. Young adults, even though not the typical at-risk population for the most lethal viral threats nonetheless seem to have the most mental struggles during the lockdown. The heightened vulnerability of this age group to mood and anxiety disorders in general and more notably in times of pandemics might be due to the fact that they might live in precarious conditions, worry about their future, and have unmet needs for peer interactions and dating [47]. Mental health was mostly shown to be negatively influenced by job security. The interplay between psychological, financial, and medical statuses is particularly relevant in a country where rising economic adversities have left a large segment with high unemployment rates, below the poverty line [50]. On one hand, those who are jobless or have a low income experience more stress during the pandemic [15] and on the other, those with lower anxiety of contacting the virus, were shown to have inferior concerns about losing their jobs [53]. Either way, the increased daily COVID-19-related stressors in under-resourced populations have been well documented [54].

Most importantly, our study adds evidence to the role of sleep and gratitude as protective factors against stress, anxiety, depression, and PTSD above and beyond gender, age, and job status in times of COVID-19. It importantly demonstrated the robust capacity of these simple behavioral tendencies to mitigate prominent mental health impact in a society battling medical and socio-political calamities at once. In terms of sleep hygiene, we found people go to bed later and sleep less well with only 50% of all participants getting the recommended 7–8 h of sleep per night, with 55% of them going to bed after midnight, and around 20% staying up past 2AM. According to Marelli et al., this delay in sleep seemed to be more prominent in college students, and correlated with a worsening sleep quality [55]. Fitbit reports indicate a comparable global shift in sleep patterns in comparison with pre-outbreak, with delayed wake-up times [56] and a culminating 40% negative changes in sleep, a 50% sleep difficulty [57] overall associated with increased levels of depression and anxiety during the pandemic [58]. We indeed found that those who with worst sleep quality had lower levels of mental health with increased severity of stress, depression, anxiety, and PTSD. While circadian rhythmicity is a cornerstone of psychological but also immune system optimization, these findings call to factor sleep regulation in addition to other broader aspects of people’s lives and health when advocating COVID-19 prevention and treatment options [59].

The Lebanese people although facing grandiose accumulating adversities are nonetheless known for their high levels of spirituality and religious commitment and as such gratitude could show culturally relevant promises. Gratitude was previously studied in non-crises [60] in addition to war-exposed populations [61] and was found to negatively correlate with PTSD, depression, burnout, and suicidality. It was correlated to adaptive coping and well-being at the onset of the pandemic [27]. We have further documented its role as a predictor of mental wellness in a tormented culture. Research had posited that gratitude promotes cognitive openness and prosocial proclivity needed to better adjust to viral-imposed lockdowns and routine alterations [62], whereby hand-washing and social distancing might no longer be viewed as punitive self-survival methods, rather as active communal safeguarding, thus lessening the negative impact of COVID-19 on one’s mental health. Gratitude could counter-balance COVID-19-induced existential vulnerability by restructuring our views on life and enjoying the small things in life [63], further shifting our mindsets from “it has stopped our lives” to “spending more time together and strengthening bonds” [64]. Future studies should subsequently investigate these protective factors in correlation with the capacity to grow post-pandemic and the ability of sleep and gratitude factors, not necessarily to dampening the overall stress symptoms, but to bring benefits of post-traumatic growth [65].

### Limitations

This study was conducted rapidly at the end of the first lockdown, at what is now considered the beginning of a two-year ongoing pandemic and relied mostly on convenience sampling of computer literate individuals and used self-rating scales for the assessment of psychological distress. Due to local quarantine guidelines, it did not include in person diagnostic tools and did not factor pre-existing mental conditions of participants. It is also difficult to separate contribution of COVID-19 in rising levels of mental distress in the country from other ongoing hardships including the financial distress. These inputs differentially influence various psychological factors as shown in concurrent comparative studies with Lebanese more stressed but less depressed than other countries such as Italy and Portugal [66]. Lastly, sleep and gratitude were subjectively retrospectively assessed. Future studies should look into prospective relationships between sleep quality/quantity, practice of gratitude and mental health outcomes.

## Conclusion

Evidence of the psychological long-term effect of the COVID-19-related restrictive measures point to a ravaging mental health harmful curve especially in a developing country in times of socio-economic turmoil. Peaking levels of stress, anxiety, depression, and mostly PTSD have especially invalidated the youth. As such ongoing evaluation of the impact of lifestyle changes associated with the pandemic is needed, notably with this at-risk population [67]. This also prompts an open dialogue between mental health experts and public officials to better protect this vulnerable group, and customize a strategic outreach to better address their psychosocial needs [68]. Improving sleep and practicing gratitude in such a cultural context of accumulating adversities, could better equip vulnerable individuals worldwide in general and in Lebanon in particular to navigate the demanding sanitary, political, and financial situation and help face the expected rise in mental health distress due to the chronicity and severity of the hardships. These positive behavioral approaches could override the scarcity of resources and lack of governmental planning in any given country.

Heightened trauma, anxiety, and depression were indeed curbed by positive health-related behaviors like sleep and gratitude. Psychological resilience in the face of the pandemic seems related to simple modifiable factors [68] and health-promotion strategies directed at adopting or maintaining those protective activities and attitudes should be further amplified to flatten the mental health curve during and after this pandemic, and potentially upcoming ones. Policy-makers and practitioner could also re-shift the focus from threats and loss towards opportunities and environmental recovery.

## Data Availability

The datasets generated and/or analyzed during the current study are not publicly available due to institutional ethics regulations but are available from the corresponding author on reasonable request.

## Abbreviations

GAD-7: Generalized anxiety disorder
GQ: Gratitude Questionnaire
IES: Impact of Events Scale
MOPH: Ministry of Public Health
PHQ-9: Patient Health Questionnaire
PSS: Perceived Stress Scale
PSQi: Pittsburg Sleep Questionnaire
PTSD: Post-traumatic stress disorder
SD: Standard deviation
WHO: World Health Organization
